# The National Association of Dental Faculties

**Published:** 1902-09

**Authors:** 


### THE NATIONAL ASSOCIATION OF DENTAL FACULTIES.

This body met at Niagara Falls, July 23, with a full representation, from New England to California.

This Association was originally very exclusive, and the dental profession had but a limited knowledge of its operations, and, in consequence, was indifferent as to the result of its work. As dental colleges multiplied in numbers and power, through their connection with the great educational systems of the country, there came an increased desire to understand the methods and the work of this organization. By some it has been regarded as a dental educational trust; by others, as a body meeting annually as a mutual admiration society, but by neither of these as an association with but one aim,—that of advancing the standard of dental education in this country.

The object of those who organized it in 1884 was made clearly evident by their action at the initial gathering. The unsettled condition of dental education at that time, with its loose methods, forced this organization into existence, and through its persistent efforts the quality as well as the reputation of the dental profession has been continually advanced.

The success that followed the original efforts induced a number of the medical colleges to attempt a similar organization, and this has been carried forward with some success, but the difficulties attending a unity of effort in medicine are far greater than in dentistry, and the result has, therefore, not been as far-reaching as that effected by the Dental Faculties Association.

It is now eighteen years since this body was launched on a troubled sea. During this period its work is represented in the union of all the dental colleges of the United States and Canada; the establishment of a broad curricula comprising all the principal foundation studies in medicine; the extension of the entrance examination to cover a liberal preparatory examination; and the increase of the period of study to four years. It is not probable that the limit of advancement has been reached, but from time to time suggested improvements will be adopted.

The session just closed was not remarkable for any special legislation. This was not anticipated. The routine work of the organition, although important and interesting to members, contained nothing of interest to the general profession. There is probably no organization, outside of State and national legislative bodies, that requires more tact to manage than this one. The marked individuality of character that prevails in the membership makes the duty of presiding officer no sinecure, and few have been found equal to its exacting duties. Appearances are, however, often deceptive, and this is true here, for out of much confusion of tongues, and oftentimes acrimonious debate, there has resulted the marked advances to which previous allusion has been made.

The Foreign Relations Committee presented its annual report, but this year it was divested of its usual number of bristling orders; in fact, was practically a repetition of those of preceding years. Heretofore, and noticeably last year, it laid down a series of laws for the government of dental colleges in their relation to foreign dental schools and foreign graduates. Its assumptions were of such a dictatorial character that some of the schools refused absolutely to enforce them, contending that they had never been legally adopted by the Association. This opposition became so formidable that these rules were laid over for future consideration. This committee has accomplished some good work, but it has become a source of constant irritation and has practically made the Association of Faculties a body secondary to its orders and that of the Foreign Advisory Board. The chairman and several members of this committee are, at this writing, in attendance at the conventions in Stockholm. It is hoped that they may absorb some wisdom in relation to international affairs, a knowledge much needed. That they will be able to bring about an harmonious adjustment in the methods of dental training between the various countries is too much of a Utopian idea to be even considered. That the social mingling may be beneficial there can be no question, but until national peculiarities change this cannot go farther than an interchange of courtesies. It is thought that the Foreign Relations Committee and the delegates from the Faculties Association will return home with an assorted cargo of words and with some ideas, but with no formulated plans for a reciprocal curriculum.

Whether the chairman of the Foreign Relations Committee will retain that position the coming year is somewhat uncertain. He declared positively that he would resign. If this is carried out, it will require a wise judgment to fill the place, with its responsible duties. Some of the higher dental schools have had a sufficiency of dictation, and if the Association of Faculties is to remain a united body all its committees must be made to understand that they are servants and not masters of the organization. When this lesson is thoroughly absorbed, the Association of Faculties will become in the future a power for good in dental educational circles, as it has been in the past.

